# Spatial-frequency complementary fusion network for dehazing with multi-scale and attention modules

**DOI:** 10.1038/s41598-026-47027-2

**Published:** 2026-04-09

**Authors:** Chenguang Yan, Gang Liu

**Affiliations:** 1 College of Applied Mathematics, Chengdu University of Information Technology, Chengdu, 610225 China; 2 Key Laboratory of Mathematical Meteorology, Chengdu University of Information Technology of Sichuan Province, Chengdu, China

**Keywords:** Image dehazing, Spatial-frequency multi-scale module, Spatial-frequency complementary attention, Feature fusion, Engineering, Mathematics and computing

## Abstract

Single image dehazing is a challenging ill-posed problem. It aims to estimate the latent haze-free image from the observed hazy image. In recent years, learning-based methods have demonstrated their superiority in single image dehazing. However, most existing learning-based dehazing methods focus exclusively on spatial-domain features, largely overlooking frequency-domain information. To address this limitation, a novel end-to-end Spatial-Frequency Complementary fusion Network is proposed for single image dehazing. Its core idea is fusing complementary frequency-domain and spatial-domain information. To efficiently incorporate frequency-domain information, the network includes two meticulously designed modules: the Spatial-Frequency Multi-scale Module and the Spatial-Frequency Complementary Attention. The former achieves deep complementary fusion of spatial- and frequency-domain features through a branched architecture, strengthening feature representation and preserving image details. The latter modulates attention-enhanced spatial features in the frequency domain and employs an adaptive gating mechanism to emphasize informative regions, thereby enabling differentiated optimization of frequency-domain features and improving dehazing performance. Extensive experiments on synthetic and real-world datasets demonstrate that our method achieves competitive results, with notably better performance in color fidelity and detail retention.

## Introduction

Single image dehazing has received considerable attention from both academia and industry in recent years. The scattering effect caused by atmospheric haze particles induces severe image degradation, primarily manifested as contrast attenuation and color deviation^1,2^. These degradations not only impact environmental monitoring and remote sensing analyses^3^, but also significantly degrade the performance of subsequent high-level vision tasks such as detection, tracking, and classification. Therefore, image dehazing, as a low-level visual task, is widely recognized as a necessary preprocessing step for high-level visual tasks. Its core objective is to recover clear images from degraded inputs.

Based on the principles of haze formation, the atmospheric scattering model was developed to remove haze from images^4–6^. It can be formally expressed as follows.

1 $$\begin{aligned} \textrm{I}(x) = \textrm{J}(x)t(x) + \alpha (1 - t(x)), \end{aligned}$$

where $\textrm{I}(x)$ is the observed hazy image, $\textrm{J}(x)$ is the true scene radiance, $\alpha$ is the global atmospheric light, and *t*(*x*) is the medium transmission map related to both scene depth and atmospheric scattering coefficient. The goal of single image dehazing is to recover the unknown $\textrm{J}(x)$, *t*(*x*), and $\alpha$ from observed $\textrm{I}(x)$ using Eq. (1). This is a ill-posed problem because the *t*(*x*) depends on unknown scene depth. Therefore, appropriately estimating atmospheric light and the transmission map is key to recovering haze-free images.

Early dehazing methods were developed based on the atmospheric scattering model and relied on various prior assumptions, such as dark channel prior (DCP)^7–9^, color-lines prior (CLP)^10^, non-local prior (NLP)^11^, color attenuation prior (CAP)^12^ and rank-one prior (ROP)^13^. Relying on handcraft priors or assumptions derived from statistical analysis, these methods^7–13^ achieve good dehazing performance but fail to adapt well to various complex scenarios.

Recently, with the rapid advancement of deep learning technology, dehazing methods^14–17^ based on convolutional neural networks (CNNs) have shown significant advantages. Benefiting from the powerful feature extraction capability of convolutions^18–20^, CNNs can effectively learn various local features from images, enabling them to distinguish between haze characteristics in complex environments and the true scene radiance. This capability leads to a significant improvement in the robustness and generalization ability of CNN-based dehazing methods. Indeed, this research paradigm, which leverages the powerful learning capability of deep learning networks to achieve adaptive processing and replace traditional fixed-parameter models, has been successfully extended from image dehazing to other related low-level vision tasks such as image denoising, yielding a series of innovative results^21,22^ and further demonstrating the broad potential of deep learning in low-level vision applications.

Early dehazing networks^14,23^ still rely on the atmospheric scattering model to recover haze-free images. They estimate the global atmospheric light and the transmission map, respectively. Then, AOD-Net^15^ re-formulated the atmospheric scattering model and adopted an all-in-one estimation strategy. Recent some methods^24–27^ demonstrate the effectiveness of end-to-end frameworks, particularly when integrated with attention mechanisms^28–30^, leading to significant performance improvements.These methods have achieved more superior and robust dehazing results.

**Fig. 1:**
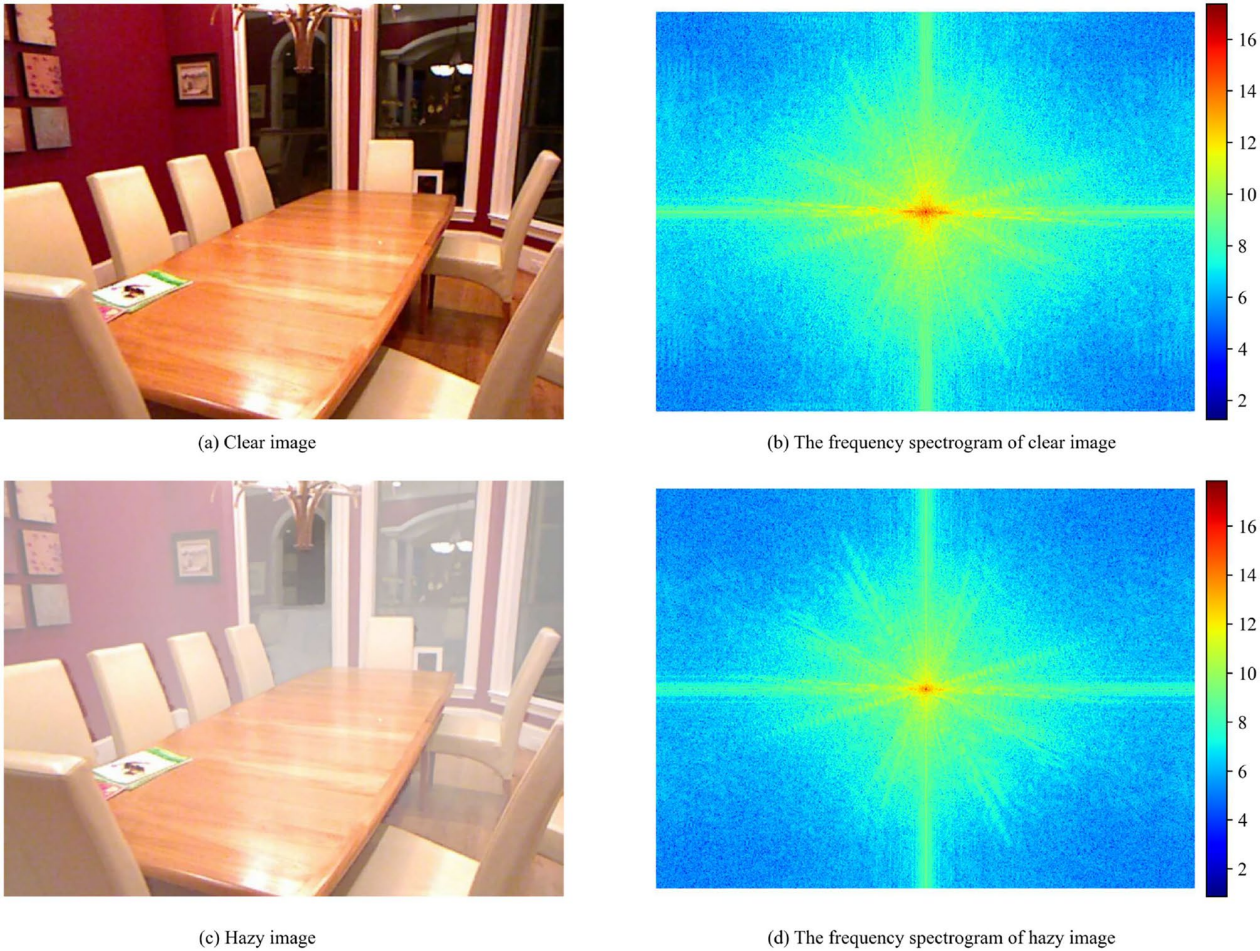
The Comparison of spectrograms between clear and hazy images. (**a**) Clear image; (**b**) The frequency spectrogram of clear image; (**c**) Hazy image; (**d**) The frequency spectrogram of hazy image.

However, these CNN-based dehazing methods are limited to processing spatial domain information only. By designing complex encoder–decoder structures^31^, residual modules^32^, or attention mechanisms^33^, they optimize the dehazing performance at the pixel level. Despite achieving significant improvements in dehazing performance, these approaches often neglect the importance of frequency-domain information. In fact, compared with spatial-domain processing, the differences between hazy and clear image pairs in the frequency domain are physically conclusive^34^. As shown in Figure 1, we visualize the spectrograms of clear images and their corresponding hazy images. It can be observed that hazy images exhibit attenuation in medium and high frequencies, along with concentrated energy in low frequencies. The high-frequency regions in images contain the detailed textures of the scene, while the low-frequency regions represent the overall brightness and background features of the scene^35^. Pure spatial domain processing methods only perform reconstruction at the pixel level and cannot effectively address the issue of frequency attenuation. Therefore, in the dehazing framework, the explicit introduction and guidance of frequency-domain information, combined with its complementary fusion with spatial-domain information, enable the network to understand the haze-scene relationship from multiple perspectives and contribute positively to achieving detail restoration with clear physical meanings. This dual-domain approach enhances the model’s ability to capture commonly neglected image features^3^. Recent methods^35–44^ have extracted frequency-domain information to address various visual tasks. For example, FSCMF^43^ proposes a dual-branch spatial-frequency joint perception network for visible and infrared image fusion, which enhances feature representation by collaboratively modeling spatial structures and frequency characteristics, demonstrating the effectiveness of joint spatial-frequency modeling in cross-domain tasks; STWANet^44^ introduces a spatio-temporal wavelet attention aggregation mechanism for remote sensing change detection, which integrates wavelet-based frequency representations with attention modeling to enhance feature aggregation. This work highlights the advantages of combining frequency-domain information with attention mechanisms in remote sensing tasks; LFENet^45^ propose a low-light stereo image enhancement and de-noising method implemented in a low-frequency information enhanced image space, which fuses low-frequency information mining with cross-view and cross-scale feature interaction to mitigate noise interference and improve feature encoding performance. This work verifies the superiority of constructing a low-frequency enhanced image space and conducting multi-dimensional feature interaction for low-light stereo vision tasks. Despite its successful application in other vision tasks, the introduction of frequency domain information remains an underexplored area in image dehazing. For instance, in the field of single image dehazing, there are relatively few dedicated feature extraction modules for frequency-domain representations; conventional attention mechanisms also rarely leverage spectral information to dynamically recalibrate the significance of distinct frequency components.

Based on the above observations and conclusions, we propose SFC-Net (Spatial-Frequency Complementary fusion Network), a novel end-to-end architecture for single image dehazing. The network adopts a U-Net-like framework. Unlike existing mainstream frequency-domain dehazing methods that adopt a “single-frequency component + sequential processing” framework, SFC-Net integrates complementary information from both the frequency and spatial domains during feature learning, focusing on the collaborative utilization and fusion of spatial-frequency features, and performing single-image dehazing from a novel perspective. To better integrate frequency-domain information, we propose several key architectural modules. These modules include the novel encoder-decoder modules, Spatial-Frequency Complementary Attention module(SFCA), and Spatial-Frequency Multi-scale feature extraction module(SFMM). Specifically, the encoder–decoder module comprises multiple novel residual blocks. It can effectively enhance the network’s feature encoding and decoding capabilities; the SFCA module integrates the attention mechanism with frequency-domain feature modulation. Unlike conventional attention modules limited to channel or spatial recalibration, the SFCA adaptively fuses spatial key-region cues and complementary frequency-domain characteristics, effectively enhancing features and improved detail preservation in single-image dehazing; the SFMM module extracts multi-scale features and, through a branched design, fuses spatial and frequency-domain features for synergistic enhancement, improving feature representation and robustness in complex haze scenarios. Extensive experiments on public datasets show that the proposed method achieves excellent dehazing performance compared to recent methods. In summary, the contributions of our work are as follows:

1. We propose a Spatial-Frequency domain Multi-scale Module (SFMM) to enhance feature extraction. This module achieves inter-branch feature complementarity through a multi-branch feature enhancement and fusion strategy, enabling deep complementary fusion of important multi-scale spatial-domain features and frequency-domain features. The multi-scale design also enables more comprehensive feature extraction, boosting the clarity and visual quality of dehazed images.
2. We propose a novel Spatial-Frequency domain Complementary Attention (SFCA). By integrating attention modeling with frequency-domain feature modulation, SFCA enables the network to focus on key spatial regions while effectively capturing frequency components that are difficult to model in the spatial domain, overcoming the limitations of single-domain processing. Moreover, the adaptive gating mechanism enables the attention module to achieve differential optimization of frequency-domain features. As a result, SFCA enhances multi-dimensional feature representation and improves dehazing performance.
3. We innovatively integrate frequency-domain information into the network architecture. By combining SFMM and SFCA, and using new residual convolutional block, a novel Spatial-Frequency Complementary fusion Network (SFC-Net) is proposed for reconstructing high-quality haze-free images. SFC-Net effectively emphasizes frequency-domain information and achieves complementary fusion with spatial-domain information across all stages of feature learning.
4. Experimental results on public datasets demonstrate that the proposed method not only achieves competitive quantitative performance but also delivers superior visual quality through enhanced color fidelity and detail preservation. Ablation studies also validate the effectiveness of each proposed module and the superiority of the network.

## Methods

**Fig. 2:**
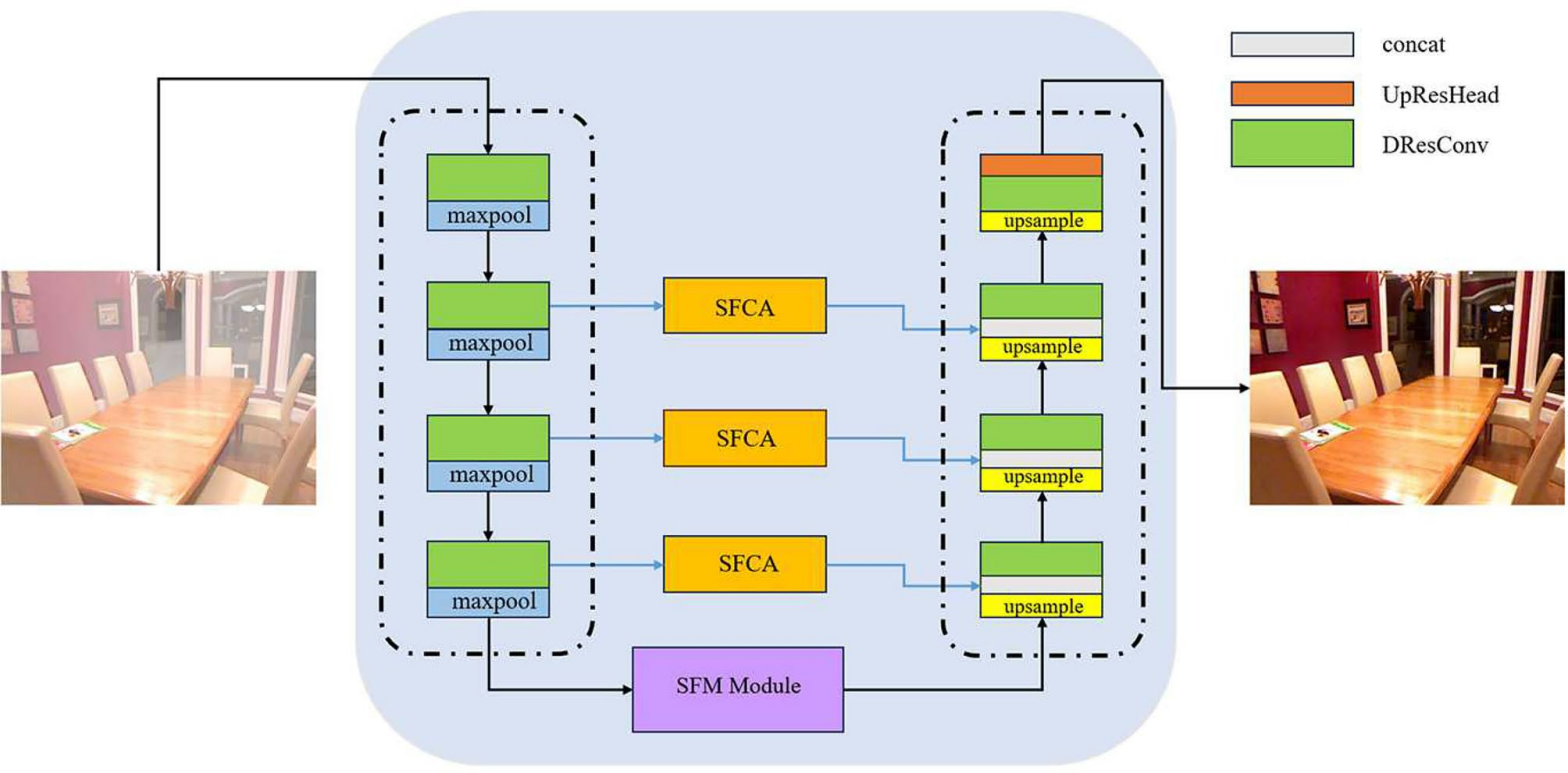
The overall architecture of our Spatial-Frequency domain Complementary Network (SFC-Net). The network is a five-layer end-to-end U-Net-like architecture, comprising three main parts: the encoder, decoder, and feature enhancement modules.

In this section, we detail our proposed SFC-Net and its associated core modules. As illustrated in Fig. 2, Our SFC-Net is an end-to-end U-net-like framework, consisting of three parts: encoder part, decoder part, and feature enhancement part. Specifically, for a hazy input image $I\in \mathbb {R}^{3\times H\times W}$, the network first processes it through the encoder, to extract hierarchical multi-scale features. In the feature enhancement stage, the SFMM(spatial-frequency multi-scale module) is adopted to enhance feature learning. During decoding, SFCA (spatial-frequency complementary attention) hierarchically integrates enhanced features from corresponding encoder levels, thereby improving the network’s representational capacity and preserving more scene details. The goal of SFC-Net is to directly restore the corresponding haze-free image $J\in \mathbb {R}^{3\times H\times W}$. The following subsections detail the core components of the SFC-Net.

### Spatial-frequency multi-scale module

**Fig. 3:**
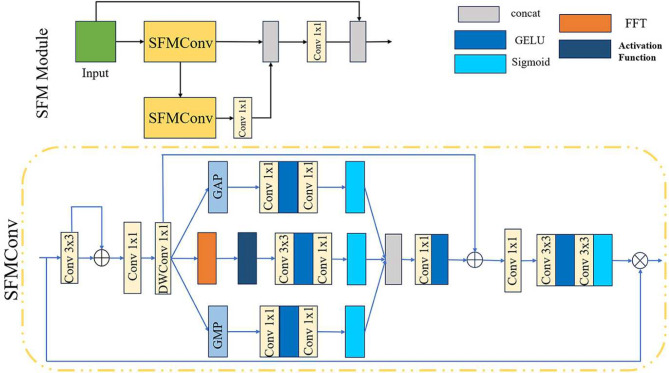
The Structures of the Space-Frequency Domain Multi-scale Module (SFMM) and Space-Frequency Domain Multi-scale Convolution (SFMConv). The detailed architectural designs of the key component in SFC-Net that enable effective spatial-frequency domain feature fusion at multiple scales.

Feature enhancement is the critical stage for learning in image dehazing networks. However, traditional multi-scale feature extraction methods in this stage have primarily focused on information at varying spatial resolutions. Through special designs such as pyramid structures^46^, dilated convolutions^47^, or convolutional layers with different strides^23^, information from local details to global structures in images can be captured. However, the introduction of frequency-domain information can overcome the limitation of relying solely on the spatial dimension, facilitating richer feature learning. Therefore, we design the SFMM in the feature enhancement part. As the core of feature enhancement, the SFMM is composed of basic spatial-frequency multi-scale convolutions (SFMConv). It achieves feature enhancement by performing feature extraction and fusion at different scales. The SFMM and SFMConv are structured as shown in Fig. 3. The SFMConv is a composite convolution unit that enhances feature representation capability by means of multi-path feature processing and attention mechanisms. Specifically, a 3 $\times$ 3 convolutional layer is first employed to adjust the channel number of the input feature and complemented by a residual connection. Then, the number of feature channels is increased via 1 $\times$ 1 convolution, followed by depthwise convolution for spatial feature extraction to reduce the number of parameters while enhancing feature expression.

2 $$\begin{aligned} \mathrm {F_{in'}} = \textrm{DWConv}(\textrm{Conv}_{1\times 1}^\textrm{up}(\mathrm {F_{in}}+\textrm{Conv}_{3\times 3}(\mathrm {F_{in}}))). \end{aligned}$$

The obtained features are processed via a multi-path approach, forming three branches. For the first branch, global average pooling is employed to capture global cross-channel dependencies. The second branch uses global max pooling to extract the most activated regions in the feature map. The third branch conducts feature learning in the frequency domain, capturing texture and structural information to compensate for the limitations of spatial-domain information. Notably, we adopt logarithmic operation $F=\log (1+|\mathcal {F}(x)|)$ to compress the dynamic range of the amplitude spectrum for activation after performing FFT. Branch outputs activated by sigmoid ($\sigma$) are concatenated, fused via a 1 $\times$ 1 convolution to generate attention weights. These weights are then combined with the original features through a residual connection.

3 $$\begin{aligned} w_\textrm{c} =&\sigma (\textrm{Conv}_{1\times 1}(\text {GELU}(\textrm{Conv}_{1\times 1}(\text {GAP}(\mathrm {F_{in'}}))))),\end{aligned}$$

4 $$\begin{aligned} w_\textrm{s} =&\sigma (\textrm{Conv}_{1\times 1}(\text {GELU}(\textrm{Conv}_{1\times 1}(\text {GMP}(\mathrm {F_{in'}}))))),\end{aligned}$$

5 $$\begin{aligned} w_\mathrm {f^{'}} =&\log {(1+|\text {FFT}(\mathrm {F_{in'}})|)},\end{aligned}$$

6 $$\begin{aligned} w_\textrm{f} =&\sigma (\textrm{Conv}_{1\times 1}(\text {GELU}(\textrm{Conv}_{3\times 3}(w_\mathrm {f^{'}})))),\end{aligned}$$

7 $$\begin{aligned} \mathrm {F_{n''}}=&\text {GELU}(\textrm{Conv}_{1\times 1}(\textrm{Concat}(w_\textrm{s}, w_\textrm{c}, w_\textrm{f})))+\mathrm {F_{in'}}. \end{aligned}$$

This design avoids excessive focus on local features at the expense of global context, while highlighting critical information. Following dimensionality reduction, secondary feature calibration is conducted to further refine feature representation. This process enables the model to focus on the most critical regions, generate weights for secondary weighting, and output enhanced features.

8 $$\begin{aligned} \mathrm {F_{out}}=\textrm{Conv}_{3\times 3}(\text {GELU}(\textrm{Conv}_{3\times 3}(\textrm{Conv}_{1\times 1}^{\textrm{down}}(\mathrm {F_{n''}}))))\mathrm {F_{in'}}. \end{aligned}$$

In this module, the GELU (Gaussian Error Linear Unit) is employed to facilitate a smoother nonlinear transformation, replacing the ReLU.

The SFMM employs three parallel branches with distinct attention mechanisms to achieve multi-scale feature enhancement. Each branch guides the model to focus on different salient regions, followed by adaptive feature fusion. This architecture enables the network to adaptively balance weights of spatial and frequency domain information. The multi-scale hierarchical learning mechanism of SFMM enhances the network’s adaptability to complex hazy scenes. These designs compensate for the flaw of traditional schemes in ignoring frequency-domain information, better preserves details, and enhances the clarity and visual quality of dehazed images.

### Spatial-frequency complementary attention

**Fig. 4:**
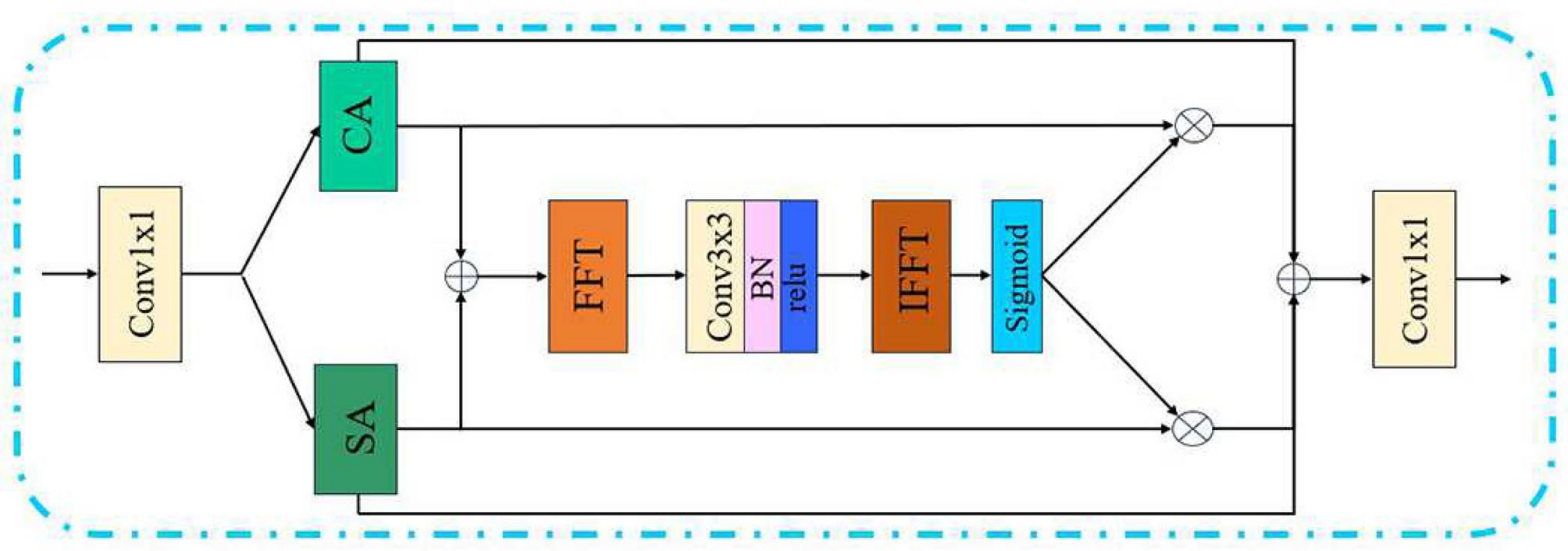
The Structure of the Spatial-Frequency Complementary Attention Module (SFCA). The attention mechanism designed to selectively emphasize important features from both spatial and frequency domains, enhancing the network’s ability to focus on haze-relevant information.

Following feature enhancement, the data flow proceeds to the decoding stage, where an attention mechanism is introduced to strengthen feature representation. Fusing features from the decoder with those from the encoder is an effective trick in deep learning-based methods. This fusion can enhance the flow of features and effectively reduce the loss of feature information during transmission. Given these trick, we propose a novel attention mechanism to enhance encoder features. The detailed structure of the attention module is illustrated in Fig. 4. Specifically, this attention mechanism retains the core ideas of the complementary fusion between frequency-domain and spatial-domain information. By employing spatial attention (SA) and channel attention (CA), the module extracts enhanced spatial features with different focus regions. These features are then fed into the frequency domain via Fourier transform for feature modulation. Spatial weights *w* are computed during this process, and the modulated features are finally fused with the original spatial features through weighted summation. Additionally, we employ skip connections to add original features to the fused features, thereby alleviating learning difficulty and preventing excessive information loss. Finally, the above information is fused through a 1 $\times$ 1 convolution to obtain the final features. This design fully leverages the advantages of frequency domain analysis to capture the global structural information of images. Meanwhile, by incorporating spatial and channel attention, the module can adaptively focus on different regions and feature channels, thereby improving dehazing performance and image quality.

9 $$\begin{aligned} \mathrm {F_{SA}}&= \text {SA}(\textrm{Conv}_{1\times 1}(\mathrm {F_{in}})),\end{aligned}$$

10 $$\begin{aligned} \mathrm {F_{CA}}&= \text {CA}(\textrm{Conv}_{1\times 1}(\mathrm {F_{in}})),\end{aligned}$$

11 $$\begin{aligned} \mathrm {F_{out}}&= \textrm{Conv}_{1\times 1}(\mathrm {F_{SA}} + \mathrm {F_{CA}} + \mathrm {F_{SA}}\cdot {w} + \mathrm {F_{CA}}\cdot {(1-w)}). \end{aligned}$$

Additionally, features at different levels contain distinct information. Incorporating attention modules at multiple levels can more efficiently improve dehazing performance. Therefore, the SFCA is incorporated at multiple encoder-decoder levels, enhancing feature representations. This leads to improved dehazing performance and better visual quality of the output images.

### DResConv and UpResHead

Besides the two core modules mentioned above, SFC-Net also includes newly designed fundamental convolutional units and an upsampling module. To effectively enhance the network’s feature extraction and information encoding capabilities, the encoder-decoder backbone is constructed with optimized dual residual convolution (DResConv) units. The DResConv architecturally integrates two novel residual convolution (ResConv) blocks and a standard convolutional layer. ResConv contains two paths: the main path extracts features through two $3\times 3$ convolutions, while the shortcut path employs a $3\times 3$ convolution to enhance feature transformation and sums the result into the main path. The activation function uses GELU. This dual-path feature fusion design enables the ResConv to extract and integrate features with distinct receptive fields, thereby allowing network to learn richer feature representations. Moreover, skip connection facilitates gradient propagation during training, effectively addressing the vanishing gradient problem. Based on the ResConv, the DResConv module can be expressed as

12 $$\begin{aligned} \mathrm {F_{out}} = \textrm{Conv}(\textrm{ResConv}(\textrm{ResConv}(\mathrm {F_{in}}))). \end{aligned}$$

**Fig. 5:**
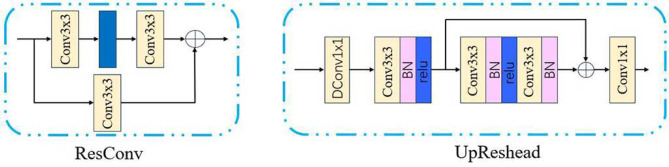
The Structures of the Residual Convolution (ResConv) and Upsample Residual Head (UpResHead). The ResConv module enhances feature extraction capabilities while facilitating gradient flow through residual connections. The UpResHead combines upsampling with residual learning to recover high-resolution features during the decoding phase.

In the final restoration stage, we avoid directly using upsampling or transposed convolution to prevent artifact generation. We design an upsampling module called UpResHead, to restore the feature maps to their original size. This module achieves upsampling through a four-layer architecture: the first layer is a deconvolution layer with a stride of 2; the second layer adopts a structure consisting of standard convolution, batch normalization (BN), and ReLU; the third layer uses a standard residual block; and the final layer employs a 1 $\times$ 1 convolution to obtain the final haze-free image. During this process, both the number of channels and the image size gradually decrease. UpResHead retains information more effectively by learning parameters for upsampling. And the strategy of gradually reducing the number of channels can significantly reduce the module’s parameter count without compromising performance. The structures of the above two modules are shown in Fig. 5.

### Overall architecture

This section concludes the description of our method by specifying the implementation details. By combining SFMM, SFCA, DResConv and UpResHead together, we propose SFC-Net, as shown in Fig. 2. The SFC-Net is a five-layer architecture. The first four layers implement feature encoding and decoding through DResconv blocks. Unlike subsequent layers using 3 $\times$ 3 convolutions, the first encoding layer specifically employs a 5 $\times$ 5 DResConv(stride = 2) to expand the receptive field. Following the DResConv, the feature maps are downsampled using max pooling. For a hazy input image $I\in \mathbb {R}^{3\times H\times W}$, the dimensional size of 1-layer, 2-layer, 3-layer, 4-layer are $64\times \frac{H}{2}\times \frac{W}{2}$, $128\times \frac{H}{2^{2}}\times \frac{W}{2^{2}}$, $256\times \frac{H}{2^{3}}\times \frac{W}{2^{3}}$, $512\times \frac{H}{2^{4}}\times \frac{W}{2^{4}}$. The final layer is a feature enhancement layer. The layer employs SFMM to produce $1024\times \frac{H}{2^{4}}\times \frac{W}{2^{4}}$ outputs. During the decoding phase, we introduce attention features from the corresponding encoder layers (4-layer, 3-layer, 2-layer), concatenate them with the decoder features, and are upsampled via transposed convolutions. Finally, the UpResHead is placed at the end of the network to generate the corresponding haze-free image $J\in \mathbb {R}^{3\times H\times W}$.

Loss function follows a similar design to the hybrid strategy in Ref.^48^, which combines Mean Squared Error (MSE) loss and Structural Similarity Index (SSIM) loss. Specifically, the hybrid loss function combines weighted pixel-level ($\mathcal {L}_{\text {MSE}}$) and structure-level ($\mathcal {L}_{\text {SSIM}}$) components, where $\mathcal {L}_{\text {MSE}}$ optimizes pixel-wise intensity consistency, while $\mathcal {L}_{\text {SSIM}}$ preserves structural details. This design aims to balance numerical accuracy and perceptual quality. The hybrid loss function is defined as

13 $$\begin{aligned} \mathcal {L}_{\text {hybrid}}=\alpha \mathcal {L}_{\text {MSE}} + (1-\alpha )\mathcal {L}_{\text {SSIM}}, \end{aligned}$$

where $\alpha$ is a hyperparameter and is empirically set to 0.7.

### Code availability

The core code of the proposed model has been uploaded to GitHub (https://github.com/lightstrider117/Spatial-frequency-complementary-fusion-network-for-dehazing-with-multi-scale-and-attention-modules.git) and will be maintained to ensure long-term accessibility.

## Results

### Datasets and metrics

In our implementation, the SFC-Net is trained and evaluated on the REalistic Single Image Dehazing (RESIDE) dataset^49^, which contains synthetic and real-world hazy images. RESIDE is a widely used public dataset for single image dehazing, comprising the Indoor Training Set (ITS), Outdoor Training Set (OTS), and three testing sets: Synthetic Objective Testing Set (SOTS), Hybrid Subjective Testing Set (HSTS), and Real-world Task-driven Testing Set (RTTS). During the training phase, for indoor scenes, 41,909 randomly cropped images from ITS are employed for training; For outdoor scenes, 72,135 randomly cropped images from OTS are employed for training. During the evaluation phase, the SOTS dataset, which is divided into two subsets (SOTS-Indoor and SOTS-Outdoor), is utilized to evaluate models trained on ITS and OTS, respectively. In addition, we evaluated the robustness of proposed SFC-Net on the I-Haze^50^, O-Haze^51^ datasets and real-world images.

The dehazing performance is evaluated using quantitative metrics, including Peak Signal-to-Noise Ratio (PSNR) and Structural Similarity Index (SSIM)^52^, which are widely used to measure the image quality. PSNR can be expressed as

14 $$\begin{aligned} \text {PSNR} = 10 \times \log _{10}\left( \frac{\text {MAX}_I^2}{\text {MSE}}\right) , \end{aligned}$$

where ${\text {MAX}_I}$ represents the maximum pixel value of the image and MSE denotes the mean square error between the predicted image and the ground-truth image. A higher PSNR value indicates lower image distortion. SSIM^52^ can be expressed as

15 $$\begin{aligned} \text {SSIM}(I,J) = \frac{(2\mu _I\mu _J + C_1)(2\sigma _{IJ} + C_2)}{(\mu _I^2 + \mu _J^2 + C_1)(\sigma _I^2 + \sigma _J^2 + C_2)}, \end{aligned}$$

where $\mu _I$ and $\mu _J$ are the means of images *I* and *J*, $\sigma _I, \sigma _J$ represent the standard deviations, $\sigma _{IJ}$ denotes the covariance, and $C_1,C_2$ are constants to avoid division by zero. The SSIM ranges from 0 to 1, where a value closer to 1 indicates a higher structural similarity between images.

To address the issue that PSNR and SSIM are inapplicable when paired clear reference images are unavailable for real-world hazy images, we introduce NIQE^53^ as a no-reference objective evaluation metric. NIQE assesses image quality based on the statistical properties of natural images, without requiring a reference image. It effectively complements subjective evaluations, providing an objective measure of the naturalness and visual quality of dehazed results in real-world scenarios, thereby enabling a more comprehensive and reliable experimental assessment. A lower NIQE score indicates better image quality.

### Implementation details

Adam is employed as the optimizer for model training, with $\beta _1$ and $\beta _2$ set to default values of 0.9 and 0.999, respectively. The initial learning rate is set to 0.0001, and a cosine annealing learning rate schedule^54^ is adopted to adjust the learning rate from the initial value to $10^{-6}$. The batch size is set to 32. All experiments are implemented in PyTorch on NVIDIA A40 GPUs.

### Performance evaluation

We compare our SFC-Net with early classic dehazing methods including DCP^8^, Dehaze-Net^14^, AOD-Net^15^, GFN^55^ and recent advanced dehazing approaches including EPDN^56^, FFA-Net^17^, RefineDNet^57^, MSTN^58^, USID-Net^59^, FSAD-Net^60^, LWDA-Net^61^ on the SOTS-Indoor and SOTS-Outdoor datasets. To ensure fair comparisons, the officially released code of these methods is employed. For methods without publicly available code, they are simulated according to their original papers and retrained on the same datasets with identical implementation details.

#### Quantitative analysis

The quantitative evaluation results (PSNR and SSIM) of SFC-Net and other methods on the SOTS datasets are shown in Table 1. As demonstrated in Table 1, our method has achieved competitive performance on both indoor and outdoor test sets. Specifically, SFC-Net achieves 30.30 dB in PSNR and 0.9513 in SSIM on SOTS-Indoor, while attaining 29.74 dB in PSNR and 0.9290 in SSIM on SOTS-Outdoor, demonstrating overall performance comparable to MSTN. Notably, on SOTS-Outdoor, the proposed method’s PSNR surpasses MSTN by 1.63 dB. This is because the incorporation of frequency-domain information enables the model to preserve finer details in more complex scenarios such as outdoor environments.

In addition, we further analyze the computational efficiency of SFC-Net in terms of runtime, the number of floating-point operations(FLOPs) and parameters(Params.), as presented in Table 1. FLOPs and runtime are measured on color images with 256 $\times$ 256 resolution. FLOPs indicate the computational complexity of the model, while Params. reflects the model’s spatial complexity. As shown in Table 1, the runtime of SFC-Net is 0.0119, which outperforms that of MSTN and FFA-Net and is only slightly higher than that of lightweight models AOD-Net and LWDA-Net. Notably, SFC-Net achieves a balance between performance and efficiency through a spatial-frequency domain information fusion and complementation mechanism. Although its Params. are significantly higher than those of other models, its FLOPs are only slightly higher than those of MSTN and USID-Net and much lower than that of FFA-Net, indicating better parameter utilization and less computational redundancy.

Table 2 presents the quantitative results of SFC-Net and several state-of-the-art methods on the I-Haze and O-Haze datasets. As shown in Table 2, SFC-Net achieves superior numerical results on I-Haze and O-Haze datasets compared to other SOTA methods, with PSNR values of 17.52 dB and 16.87 dB, respectively. This indicates that SFC-Net’s effectiveness is maintained when confronted with various types of hazy images, demonstrating strong generalization capability and robustness.

Table 3 presents the quantitative evaluation of SFC-Net and other methods on randomly selected real-world images. SFC-Net achieves significantly better NIQE scores than other deep learning-based methods, ranking only behind DCP. This is because traditional physics-based dehazing methods, represented by DCP, typically produce results with stronger contrast and sharper edges^62^, which are therefore favored by no-reference metrics based on the statistical properties of natural images. Overall, the proposed method demonstrates competitive performance, outperforming various mainstream deep learning algorithms and indicating its effectiveness in enhancing the visual quality and naturalness of hazy images in real-world scenarios.

**Table 1:** The quantitative evaluation results of various dehazing methods on SOTS-Indoor and SOTS-Outdoor. **Bold** indicates the best results.

Methods	SOTS-Indoor		SOTS-Outdoor		Run time(s)	FLOPs (G)	Param. (M)
	PSNR	SSIM	PSNR	SSIM			
(TPAMI’10)DCP	16.61	0.8546	19.14	0.8605	–	–	–
(TIP’16)Dehaze-Net	17.89	0.8069	20.59	0.8828	0.8711	0.5139	0.0083
(ICCV’17)AOD-Net	17.23	0.8105	19.49	0.8730	0.0027	0.1144	0.0017
(CVPR’18)GFN	22.30	0.8800	21.55	0.8444	–	–	–
(CVPR’19)EPDN	21.55	0.9071	22.32	0.8683	–	–	–
(AAAI’20)FFA-Net	29.41	0.9218	29.54	0.9226	0.2569	287.53	4.4560
(TIP’21)RefineDNet	24.23	0.9431	20.61	0.8798	–	–	–
(TMM’22)MSTN	**30.79**	**0.9729**	28.11	**0.9405**	0.0150	42.720	18.915
(TMM’23)USID-Net	21.41	0.8947	23.89	0.9190	0.1608	40.423	3.7700
(TNNLS’23)FSAD-Net	23.41	0.9336	26.38	0.9292	–	–	–
(NNICE’24)LWDA-Net	22.69	0.8756	24.57	0.8947	0.0090	15.593	0.2549
(Ours)SFC-Net	30.30	0.9513	**29.74**	0.9290	0.0119	47.538	115.04

**Table 2:** The quantitative evaluation results of various dehazing methods on I-Haze and O-Haze. Bold indicates the best results.

Methods	I-Haze		O-Haze	
	PSNR	SSIM	PSNR	SSIM
(ICCV’17)AOD-Net	15.80	0.7082	15.98	0.6187
(AAAI’20)FFA-Net	13.17	0.6461	15.51	0.6177
(TMM’22)MSTN	16.25	**0.7680**	15.71	0.6332
(TMM’23)USID-Net	16.02	0.7241	14.84	0.5630
(Ours)SFC-Net	**17.52**	0.7446	**16.87**	**0.6409**

**Table 3:** NIQE scores of different dehazing methods on real-world images. Bold indicates the best results.

Methods	Hazy image	DCP	AOD-Net	USID-Net	MSTN	(Ours) SFC-Net
NIQE	3.4489	**2.9765**	3.1908	3.2639	3.0176	2.9973

#### Qualitative analysis

**Fig. 6:**
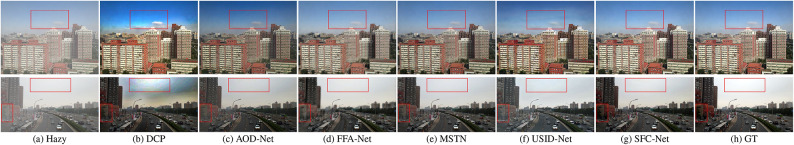
Qualitative comparison of various dehazing methods on SOTS-Outdoor dataset. (**a**) Hazy input image; (**b**–**g**) Dehazing results generated by state-of-the-art methods; (**h**) Ground truth (GT). Visual results demonstrate that our method produces images with better color fidelity, contrast restoration, and detail preservation compared to existing approaches.

**Fig. 7:**
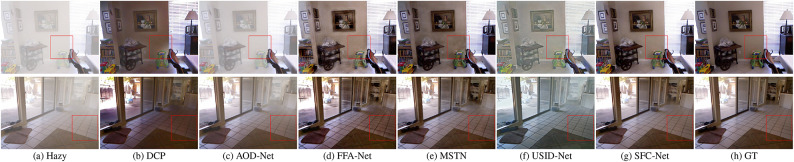
Qualitative comparison of different dehazing methods on SOTS-Indoor dataset. (**a**) Hazy input image; (**b**–**g**) Dehazing results generated by competing methods; (**h**) Ground truth (GT). Visually, our SFC-Net achieves superior color fidelity, most notably by avoiding color distortion in regions with large color blocks.

Figures 6 and 7 present the qualitative comparisons between our proposed method and several previous state-of-the-art approaches on the SOTS dataset. It can be observed that the haze-free results generated by SFC-Net exhibit superior visual quality. They retain more details while showing relatively less haze residue, showing more natural scene restoration. For example, from a comparison of the second subfigures in Fig. 6e,g, the result of SFC-Net achieves better detail preservation and haze removal effectiveness in the left region. In indoor scenes as shown in Fig. 7, the results of SFC-Net exhibit more remarkable performance in maintaining color consistency, particularly in regions with large color blocks. This performance improvement is attributed to SFC-Net’s effective mitigation of the inherent limitations in spatial-domain features through frequency-domain information integration. However, upon closer inspection, it can be observed that in regions with deep scene content, SFC-Net’s dehazing performance is not fully effective, as illustrated in the second subfigure of Fig. 7g. In the innermost corners of indoor scenes, the images remain somewhat blurred, with residual haze still visible.

**Fig. 8:**
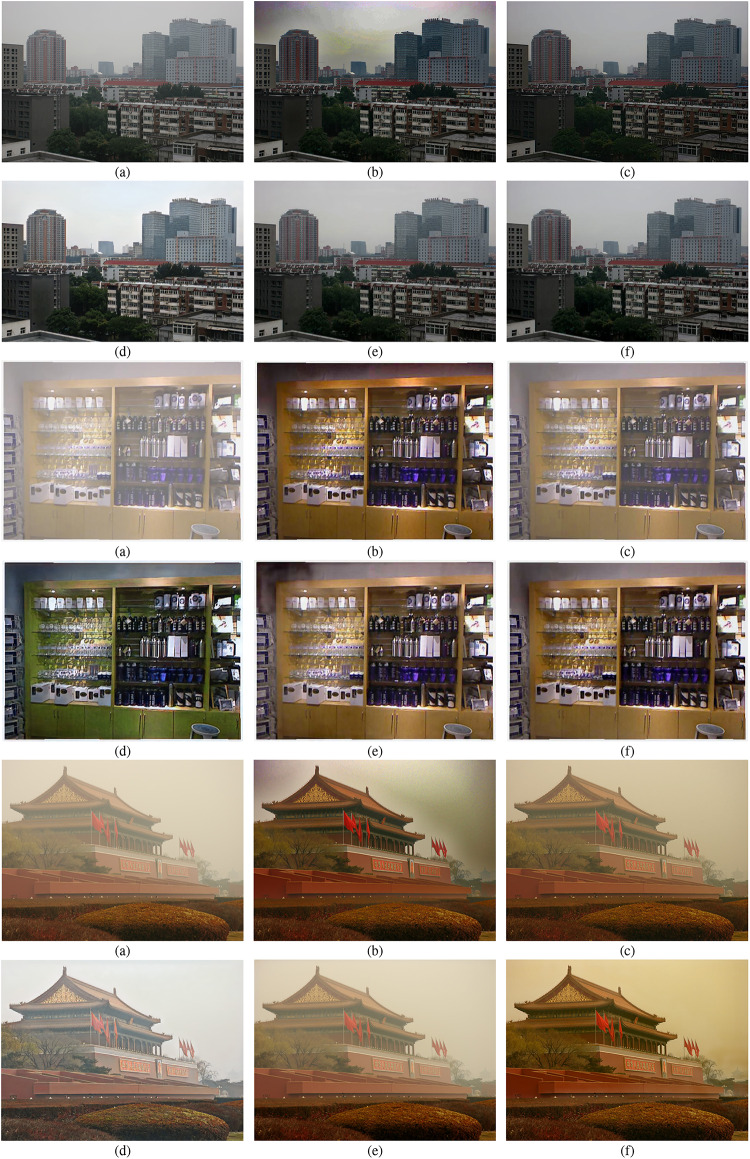
Qualitative comparison on real-world hazy images. (**a**) Hazy input image; dehazed results by (**b**) DCP, (**c**) AOD-Net, (**d**) USID-Net, (**e**) MSTN, (**f**) Our SFC-Net. It can be observed that our method yields superior outcomes in detail preservation and color fidelity within real-world scenarios.

Figure 8 further presents comparisons of dehazing results between SFC-Net and other approaches in real-world scenarios. As shown in Fig. 8, DCP and USID-Net exhibit color distortion and visual artifacts. AOD-Net exhibits noticeable haze residue, leading to unsatisfactory dehazing results. MSTN achieves remarkable performance. However, it still presents minor artifacts, such as the result of MSTN in (e) at the top-left corner of the fourth row. SFC-Net achieves excellent dehazing results, effectively removing haze while preserving fine details. However, in regions with dense haze or complex textures, slight color distortions can still be observed, as illustrated in subfigure (f) of the sixth row.

These experimental results indicate that the integrated frequency-domain information and our feature-complementary fusion design can effectively enhance the model’s capabilities in detail preservation and color fidelity.

### Ablation study

To demonstrate the effectiveness of SFC-Net, we conduct ablation experiments by considering combinations of different modules, including DResConv, UpResHead, SFMM and SFCA. The baseline is the original U-Net. M1 includes only DResConv and UpResHead; M2 adds the SFMM to M1; M3 integrates the SFCA into M1; M4 combines all modules, is the complete SFC-Net. For a more rigorous performance evaluation, tests were conducted on both subsets of SOTS (SOTS-Outdoor, SOTS-Indoor). The results are presented in Tables 4 and 5, respectively.

**Table 4:** Ablation study of SFC-Net on SOTS-Outdoor.

Model	U-Net	DResConv UpResHead	SFMM	SFCA	PSNR	SSIM
Baseline	$\surd$				18.97	0.5247
M1	$\surd$	$\surd$			23.73	0.9021
M2	$\surd$	$\surd$	$\surd$		29.13	0.9176
M3	$\surd$	$\surd$		$\surd$	29.06	0.9144
M4(SFC-Net)	$\surd$	$\surd$	$\surd$	$\surd$	$\mathbf {29.74}$	$\mathbf {0.9290}$

**Table 5:** Ablation study of SFC-Net on SOTS-Indoor.

Model	U-Net	DResConv UpResHead	SFMM	SFCA	PSNR	SSIM
Baseline	$\surd$				20.05	0.6586
M1	$\surd$	$\surd$			25.41	0.9411
M2	$\surd$	$\surd$	$\surd$		30.54	0.9387
M3	$\surd$	$\surd$		$\surd$	$\mathbf {30.59}$	0.9506
M4(SFC-Net)	$\surd$	$\surd$	$\surd$	$\surd$	30.30	$\mathbf {0.9513}$

From Table 4, We observe that M4(SFC-Net) achieves the highest PSNR and SSIM on SOTS-Outdoor. Compared to the baseline, M1 shows a significant improvement. This indicates that DResConv and UpResHead can effectively enhance the model’s performance. After integrating the SFMM and SFCA, M2 and M3 show remarkable numerical improvements compared with M1. This further validates the effectiveness of these modules in enhancing image restoration quality and dehazing performance. The results in Table 5 also show similar conclusions for the SOTS-Indoor.

**Fig. 9:**
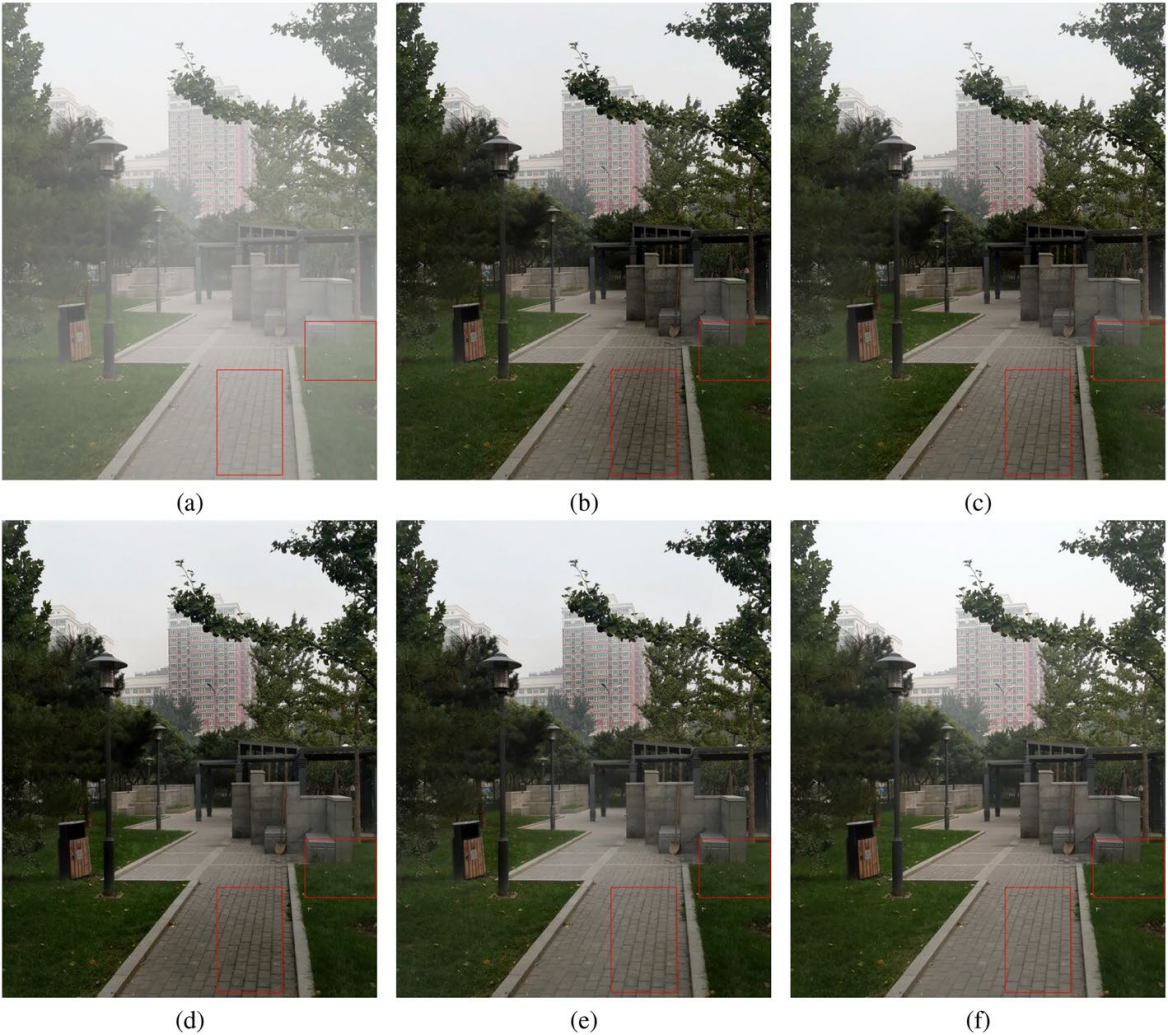
Ablation study on key components of SFC-Net. (**a**) Hazy input; dehazed results by (**b**) M1, (**c**) M2, (**d**) M3, (**e**) M4 (full model); (**f**) Ground truth. M1 includes only DResConv and UpResHead; M2 adds the SFMM to M1; M3 integrates the SFCA into M1; M4 combines all modules, is the complete SFC-Net. The progressive improvement from (**b**–**e**) visually validates the contribution of each added component.

Furthermore, we present the qualitative results of the ablation study, as shown in Fig. 9. It can be observed that M1’s dehazing image (b) exhibits color distortion and incomplete haze removal. (c) shows the result of M2. M2 reduces color distortion effectively by supplementing frequency-domain features with SFMM, though residual haze persists in local regions [e.g., near the lawn and stone pillars on the right side of (c)]. M3 effectively eliminates the residual haze in local regions, as shown in (d). This is because SFCA helps the model focus on the differences between hazy regions and the haze-free scene. However, there is still slight color distortion. (e) shows the result of our proposed SCF-Net (M4). By comparing with previous results, M4 achieves the best visual result. The combined use of SFMM and SFCA effectively removes haze while maintaining color fidelity, producing results visually closest to the ground truth (f). These experimental results demonstrate that our dual-domain complementary fusion (frequency-domain information and spatial-domain information) strategy effectively mitigates the limitations of single-domain processing. These specifically designed modules contributes positively to enhancing the model’s dehazing capability and detail preservation effect.

To rigorously verify that the performance improvement of the proposed method does not primarily result from a simple increase in model capacity, we designed a new set of ablation experiments. Specifically, we constructed a comparative baseline model (SFC-Net-Baseline) based on the U-Net architecture with a parameter scale comparable to that of the proposed model. This baseline maintains a structure highly consistent with the proposed model, fully preserving the standard encoder–decoder framework of U-Net, including the skip-connection mechanism, and employing double convolution blocks as the basic feature extraction units. The only difference is that the core feature learning and enhancement module proposed in our work is removed. Furthermore, to evaluate model efficiency and investigate potential structural redundancy, we constructed a lightweight variant (SFC-Net-Light) by halving the channel width of the original model.

**Table 6:** Ablation results for verifying the source of performance improvement bold indicates the best results.

Methods	Param.(M)	SOTS-Outdoor		SOTS-Indoor	
		PSNR	SSIM	PSNR	SSIM
SFC-Net-Baseline	124.11	24.81	0.9053	24.12	0.9285
SFC-Net-Light	28.84	28.84	0.8404	29.45	0.9096
(Ours)SFC-Net	115.04	**29.74**	**0.9290**	**30.30**	**0.9513**

The experimental results in Table 6 show that the baseline model has 124M parameters, slightly higher than the proposed method, yet it fails to reach the performance level of the proposed model across all key metrics. For example, on the SOTS-outdoor dataset, the baseline model achieves a PSNR of 24.81 dB,and an SSIM of 0.9053, all of which are inferior to the corresponding results of the proposed model (PSNR: 29.74 dB, SSIM: 0.9290). Moreover, the lightweight model still achieves a PSNR of 28.94 dB and an SSIM of 0.8404, exhibiting only slight performance degradation. The decrease in SSIM is mainly due to the reduced ability of the lightweight model, compared with the full-size model, in fine detail reconstruction and texture preservation. Overall, these results further demonstrate that the performance improvement of the proposed method primarily stems from the effectiveness of the network structure design, rather than a simple increase in parameter scale.

## Discussion

In this paper, we propose an end-to-end SFC-Net for single image dehazing. Different from conventional dehazing models that rely solely on spatial processing, SFC-Net proposes a spatial-frequency feature complementary utilization and fusion framework, which incorporates frequency-domain information into the dehazing process and integrates it with spatial-domain features in a complementary manner. This strategy allows the model to capture global structure, complementing the local details represented in spatial-domain features, providing a new pathway for enhancing image dehazing performance. Our SFC-Net consists of several key components. First, the SFMM module employs a multi-scale branched architecture to deeply integrate spatial-domain features with frequency-domain information, enhancing feature representation and improving the model’s expressive capacity. Second, the SFCA module combines channel-spatial attention to accurately highlight important regions, extracts hidden spatial features through frequency-domain transformations, and incorporates an adaptive gating mechanism to dynamically adjust the contribution of different frequency components, thereby optimizing frequency-domain features in a differentiated manner. In addition, we designed an improved residual block and a novel upsampling module: the residual block enhances feature extraction and encoding capabilities, while the upsampling module effectively reduces artifacts. Extensive experiments demonstrate that our SFC-Net achieves excellent results both quantitatively and qualitatively.

Although our model achieves competitive performance in experiments, there is still room for improvement in terms of dehazing effectiveness under extremely complex scenarios and model spatial complexity. The feature extraction capability of the SFMM module is limited in extreme conditions, such as heavy haze, where subtle target features may not be fully captured, resulting in incomplete dehazing in deeper regions. In complex texture scenarios, the attention weight allocation of the SFCA module lacks sufficient adaptability to texture details, which may lead to minor color distortions. While the targeted design of these modules enhances feature fusion, it also increases the number of model parameters. However, this increase does not significantly affect overall training or inference costs. In future work, we plan to conduct an in-depth exploration of the aforementioned optimization directions. Specifically, we aim to further improve the multi-scale feature extraction branch of the SFMM module to enhance its ability to capture subtle features, and to refine the attention weight allocation strategy of the SFCA module to increase its adaptability to complex scenarios. In addition, we intend to incorporate lightweight techniques, such as depthwise separable convolution, grouped convolution, and structural re-parameterization, and systematically evaluate their suitability within our model architecture. Through comparative experiments, we will identify the optimal solutions, striving to reduce the model’s spatial complexity while maintaining its dehazing performance.

## Data Availability

The RESIDE, O-Haze, and I-Haze datasets used in this study are publicly available. For dataset access, refer to the official repositories: RESIDE (https://sites.google.com/view/reside-dehaze-datasets), O-Haze (https://data.vision.ee.ethz.ch/cvl/ntire18/o-haze/), I-Haze (https://data.vision.ee.ethz.ch/cvl/ntire18//i-haze/).

## References

[1] Jung, C. R. Efficient background subtraction and shadow removal for monochromatic video sequences. *IEEE Trans. Multimed.* **11**, 571–577. 10.1109/TMM.2009.2012924 (2009).

[2] Tan, R. T. Visibility in bad weather from a single image. In *Proc. IEEE Conf. Comput. Vis. Pattern Recogn.* 1–8. 10.1109/CVPR.2008.4587643 (2008).

[3] He, S. et al. A distinctive eocene Asian monsoon and modern biodiversity resulted from the rise of eastern Tibet. *Sci. Bull.* **67**, 2245–2258. 10.1016/j.scib.2022.10.006 (2022). 10.1016/j.scib.2022.10.006 36546000

[4] Cantor, A. Optics of the atmosphere-scattering by molecules and particles. *IEEE J. Quantum Electron.* **14**, 698–699. 10.1109/JQE.1978.1069864 (1978).

[5] Narasimhan, S. G. & Nayar, S. K. Chromatic framework for vision in bad weather. In *Proc. IEEE Conf. Comput. Vision Pattern Recognit.*, 598–605. 10.1109/CVPR.2000.855874 (2000).

[6] Narasimhan, S. G. & Nayar, S. K. Contrast restoration of weather degraded images. *IEEE Trans. Pattern Anal. Mach. Intell.* **25**, 713–724. 10.1109/TPAMI.2003.1201821 (2003).

[7] He, K., Sun, J. & Tang, X. Single image haze removal using dark channel prior. In *Proc. IEEE Conf. Comput. Vision Pattern Recognit.*, 1956–1963. 10.1109/CVPR.2009.5206515 (2009). 10.1109/TPAMI.2010.168 20820075

[8] He, K., Sun, J. & Tang, X. Single image haze removal using dark channel prior. *IEEE Trans. Pattern Anal. Mach. Intell.* **33**, 2341–2353. 10.1109/TPAMI.2010.168 (2011). 20820075 10.1109/TPAMI.2010.168

[9] Zhu, M., He, B., Liu, J. & Zhang, L. Dark channel: The devil is in the details. *IEEE Signal Process. Lett.* **26**, 981–985. 10.1109/LSP.2019.2914559 (2019).

[10] Fattal, R. Dehazing using color-lines. *ACM Trans. Graph.* **34**, 1–14. 10.1145/2651362 (2015).

[11] Berman, D., Treibitz, T. & Avidan, S. Non-local image dehazing. In *Proc. IEEE Conf. Comput. Vision Pattern Recognit.*, 1674–1682. 10.1109/CVPR.2016.185 (2016).

[12] Zhu, Q., Mai, J. & Shao, L. A fast single image haze removal algorithm using color attenuation prior. *IEEE Trans. Image Process.* **24**, 3522–3533. 10.1109/TIP.2015.2446191 (2015). 26099141 10.1109/TIP.2015.2446191

[13] Liu, J., Liu, R. W., Sun, J. & Zeng, T. Rank-one prior: Toward real-time scene recovery. In *Proc. IEEE Conf. Comput. Vision Pattern Recognit.*, 14797–14805. 10.1109/CVPR46437.2021.01456 (2021).

[14] Cai, B., Xu, X., Jia, K., Qing, C. & Tao, D. Dehazenet: An end-to-end system for single image haze removal. *IEEE Trans. Image Process.* **25**, 5187–5198. 10.1109/TIP.2016.2598681 (2016). 28873058 10.1109/TIP.2016.2598681

[15] Li, B., Peng, X., Wang, Z., Xu, J. & Feng, D. Aod-net: All-in-one dehazing network. In *Proc. IEEE Int. Conf. Comput. Vision*, 4780–4788. 10.1109/ICCV.2017.511 (2017).

[16] Li, P., Tian, J., Tang, Y., Wang, G. & Wu, C. Deep retinex network for single image dehazing. *IEEE Trans. Image Process.* **30**, 1100–1115. 10.1109/TIP.2020.3040075 (2021). 33259301 10.1109/TIP.2020.3040075

[17] Qin, X., Wang, Z., Bai, Y., Xie, X. & Jia, H. Ffa-net: Feature fusion attention network for single image dehazing. In *Proc. AAAI Conf. Artif. Intell.*, 11908–11915, 10.1609/aaai.v34i07.6996 (2020).

[18] LeCun, Y., Bottou, L., Bengio, Y. & Haffner, P. Gradient-based learning applied to document recognition. *Proc. IEEE* **86**, 2278–2324. 10.1109/5.726791 (1998).

[19] Krizhevsky, A., Sutskever, G. E. & Hinton, G. E. Imagenet classification with deep convolutional neural networks. In *Advances in Neural Information Processing Systems (NIPS)*, 1097–1105. 10.5555/2999134.2999257 (2012).

[20] Simonyan, K. & Zisserman, A. Very deep convolutional networks for large-scale image recognition. In *Proc. Int. Conf. Learn. Represent. (ICLR)*, 1–14. 10.48550/arXiv.1409.1556 (2015).

[21] Xi, R., Lyu, J., Sun, K. & Ma, T. Learning kernel parameter lookup tables to implement adaptive bilateral filtering. *Vis. Comput.*10.1007/s00371-024-03553-6 (2024).

[22] Xi, R. et al. Learning filter selection policies for interpretable image denoising in parametrised action space. *IET Image Proc.* **18**, 951–960. 10.1049/ipr2.12997 (2024).

[23] Ren, W. *et al.* Single image dehazing via multi-scale convolutional neural networks. In *Proc. Eur. Conf. Comput. Vis. (ECCV)*, 154–169. 10.1007/978-3-319-46475-6_10 (Springer, 2016).

[24] Zhang, H. & Patel, V. M. Densely connected pyramid dehazing network. In *Proc. IEEE Conf. Comput. Vision Pattern Recognit.*, 3194–3203. 10.1109/CVPR.2018.00337 (2018).

[25] Dong, H. *et al.* Multi-scale boosted dehazing network with dense feature fusion. In *Proc. IEEE Conf. Comput. Vision Pattern Recognit.*, 2154–2164. 10.1109/CVPR42600.2020.00223 (2020).

[26] Li, Y. *et al.* Lap-net: Level-aware progressive network for image dehazing. In *Proc. IEEE Int. Conf. Comput. Vision*, 3275–3284. 10.1109/ICCV.2019.00337 (2019).

[27] Bai, H., Pan, J., Xiang, X. & Tang, J. Self-guided image dehazing using progressive feature fusion. *IEEE Trans. Image Process.* **31**, 1217–1229. 10.1109/TIP.2022.3140609 (2022). 35015639 10.1109/TIP.2022.3140609

[28] Ye, T., Liu, X., Wang, X., Zhang, H. & Yang, M.-H. Perceiving and modeling density for image dehazing. In *Proc. Eur. Conf. Comput. Vis. (ECCV), Lecture Notes in Computer Science*, 124–141. 10.1007/978-3-031-19800-7_8 (Springer, 2022).

[29] Lin, C., Rong, X. & Yu, X. Msaff-net: Multiscale attention feature fusion networks for single image dehazing and beyond. *IEEE Trans. Multimed.* **25**, 3089–3100. 10.1109/TMM.2022.3155937 (2023).

[30] Liu, Y., Yin, H., Wan, J., Liu, Z. & Chong, A. Edge aware network for image dehazing. *IEEE Signal Process. Lett.* **29**, 174–178. 10.1109/LSP.2021.3130014 (2022).

[31] Ronneberger, O., Fischer, P. & Brox, T. U-net: Convolutional networks for biomedical image segmentation. In *Proc. Med. Image Comput. Comput.-Assist. Interv. (MICCAI), Lecture Notes in Computer Science*, 234–241. 10.1007/978-3-319-24574-4_28 (Springer, 2015).

[32] He, K., Zhang, X., Ren, S. & Sun, J. Deep residual learning for image recognition. In *Proc. IEEE Conf. Comput. Vision Pattern Recognit.*, 770–778. 10.1109/CVPR.2016.90 (2016).

[33] Vaswani, A. *et al.* Attention is all you need. In *Proc. Int. Conf. Neural Inf. Process. Syst. (NeurIPS)*, 6000–6010. 10.5555/3295222.3295349 (Curran Associates Inc., 2017).

[34] Yu, H. *et al.* Frequency and spatial dual guidance for image dehazing. In *Proc. Eur. Conf. Comput. Vis. (ECCV), Lecture Notes in Computer Science*, 181–198. 10.1007/978-3-031-19800-7_11 (Springer, 2022).

[35] Cai, M. *et al.* Frequency domain image translation: More photo-realistic, better identity-preserving. In *Proc. IEEE Int. Conf. Comput. Vision*, 13910–13920. 10.1109/ICCV48922.2021.01367 (2021).

[36] Yang, Y. & Soatto, S. Fda: Fourier domain adaptation for semantic segmentation. In *Proc. IEEE Conf. Comput. Vision Pattern Recognit.*, 4084–4094. 10.1109/CVPR42600.2020.00414 (2020).

[37] Suvorov, R. *et al.* Resolution-robust large mask inpainting with fourier convolutions. In *Proc. IEEE Winter Conf. Appl. Comput. Vis. (WACV)*, 3172–3182. 10.1109/WACV51458.2022.00323 (2022).

[38] Feijoo, D., Benito, J. C., Garcia, A. & Conde, M. V. Darkir: Robust low-light image restoration. In *Proc. IEEE Conf. Comput, Vision Pattern Recognit.* (2025).

[39] Zhang, H. Attention-guided residual fourier transformation network for single image deblurring. In *Proc. Pattern Recognit. Comput. Vis. (PRCV), Lecture Notes in Computer Science*, 56–68. 10.1007/978-981-97-8692-3_5 (Springer Nature, 2025).

[40] Zou, W. *et al.* Sdwnet: A straight dilated network with wavelet transformation for image deblurring. In *Proc. IEEE Int. Conf. Comput. Vision Workshops (ICCVW)*, 1895–1904. 10.1109/ICCVW54120.2021.00216 (2021).

[41] Rao, Y., Zhao, W., Zhu, Z., Lu, J. & Zhou, J. Global filter networks for image classification. In *Proc. Int. Conf. Neural Inf. Process. Syst. (NeurIPS)*, 980–993. 10.48550/arXiv.2107.00645 (Curran Associates Inc., 2021).

[42] Shen, H. et al. Spatial-frequency adaptive remote sensing image dehazing with mixture of experts. *IEEE Trans. Geosci. Remote Sens.* **62**, 1–14 (2024).

[43] Zhang, X. et al. Dual-branch frequency-spatial joint perception cross-modality network for infrared and visible image fusion. *Neurocomputing* **641**, 130376. 10.1016/j.neucom.2025.130376 (2025).

[44] Zhang, X., Dong, K., Cheng, D., Hua, Z. & Li, J. Stwanet: Spatio-temporal wavelet attention aggregation network for remote sensing change detection. *IEEE J. Sel. Top. Appl. Earth Obs. Remote Sens.* **18**, 8813–8830. 10.1109/JSTARS.2025.3551093 (2025).

[45] Zhao, M. et al. Low-light stereo image enhancement and de-noising in the low-frequency information enhanced image space. *Expert Syst. Appl.* **265**, 125803. 10.1016/j.eswa.2024.125803 (2025).

[46] Lin, T.-Y. *et al.* Feature pyramid networks for object detection. In *Proc. IEEE Conf. Comput. Vision Pattern Recognit.*, 936–944. 10.1109/CVPR.2017.106 (2017).

[47] Yu, F. & Koltun, V. Multi-scale context aggregation by dilated convolutions. In *Proc. Int. Conf. Learn. Represent. (ICLR)*, 1–9. 10.48550/arXiv.1511.07122 (San Juan, 2016).

[48] Wu, Y., Qin, Y., Wang, Z., Ma, X. & Cao, Z. Densely pyramidal residual network for UAV-based railway images dehazing. *Neurocomputing* **371**, 124–136 (2020).

[49] Li, B. et al. Benchmarking single-image dehazing and beyond. *IEEE Trans. Image Process.* **28**, 492–505. 10.1109/TIP.2018.2867951 (2019). 10.1109/TIP.2018.2867951 30176593

[50] Ancuti, C., Ancuti, C. O., De Vleeschouwer, C. & Bovik, A. C. I-haze: A dehazing benchmark with real hazy and haze-free indoor images. In *Proc. Int. Conf. Adv. Concepts Intell. Vision Syst.*, 620–631. 10.1007/978-3-030-01449-0_52 (2018).

[51] Ancuti, C., Ancuti, C. O., De Vleeschouwer, C. & Bovik, A. C. O-haze: A dehazing benchmark with real hazy and haze-free outdoor images. In *Proc. IEEE Conf. Comput. Vision Pattern Recognit. Workshops*, 1–10. 10.1109/CVPRW.2018.00132 (2018).

[52] Wang, Z., Bovik, A. C., Sheikh, H. R. & Simoncelli, E. P. Image quality assessment: From error visibility to structural similarity. *IEEE Trans. Image Process.* **13**, 600–612. 10.1109/TIP.2003.819861 (2004). 15376593 10.1109/tip.2003.819861

[53] Mittal, A., Soundararajan, R. & Bovik, A. C. Making a completely blind image quality analyzer. *IEEE Signal Process. Lett.* **20**, 209–212. 10.1109/LSP.2012.2227726 (2013).

[54] He, T. *et al.* Bag of tricks for image classification with convolutional neural networks. In *Proc. IEEE Conf. Comput. Vision Pattern Recognit.*, 558–567. 10.1109/CVPR.2019.00065 (2019).

[55] Zhang, H. & Patel, V. M. Gated fusion network for single image dehazing. In *Proc. IEEE Conf. Comput. Vision Pattern Recognit.*, 1181–1190. 10.1109/CVPR.2019.00127 (2019).

[56] Qu, Y., Chen, Y., Huang, J. & Xie, Y. Enhanced pix2pix dehazing network. In *Proc. IEEE Conf. Comput. Vision Pattern Recognit.*, 8152–8160. 10.1109/CVPR.2019.00835 (2019).

[57] Zhao, S., Zhang, L., Shen, Y. & Zhou, Y. Refinednet: A weakly supervised refinement framework for single image dehazing. *IEEE Trans. Image Process.* **30**, 3391–3404. 10.1109/TIP.2021.3060873 (2021). 33651690 10.1109/TIP.2021.3060873

[58] Yi, Q., Li, J., Fang, F., Jiang, A. & Zhang, G. Efficient and accurate multi-scale topological network for single image dehazing. *IEEE Trans. Multimed.* **24**, 3114–3128. 10.1109/TMM.2021.3093724 (2022).

[59] Li, J., Li, Y., Zhuo, L., Kuang, L. & Yu, T. Usid-net: Unsupervised single image dehazing network via disentangled representations. *IEEE Trans. Multimed.* **25**, 3587–3601. 10.1109/TMM.2022.3163554 (2023).

[60] Zhou, Y. et al. Fsad-net: Feedback spatial attention dehazing network. *IEEE Trans. Neural Netw. Learn. Syst.* **34**, 7719–7733. 10.1109/TNNLS.2022.3146004 (2023). 35130175 10.1109/TNNLS.2022.3146004

[61] Hua, Z., Hua, Z. & Li, J. Lwda-net: A lightweight dual-attention network for single image dehazing. In *Proc. Int. Conf. Neural Networks, Inf. Commun. Eng. (NNICE)*, 415–420. 10.1109/NNICE61279.2024.10499091 (2024).

[62] Li, Z., Zheng, C., Shu, H. & Wu, S. Dual-scale single image dehazing via neural augmentation. *IEEE Trans. Image Process.* **31**, 6213–6223. 10.1109/TIP.2022.3207571 (2022). 36149996 10.1109/TIP.2022.3207571

